# Orthopedic Treatment of Pycnodysostosis: A Systematic Review

**DOI:** 10.7759/cureus.24275

**Published:** 2022-04-19

**Authors:** Taha M Taka, Brandon Lung, Hayk Stepanyan, David So, Steven Yang

**Affiliations:** 1 Orthopaedic Surgery, University of California Riverside, Riverside, USA; 2 Orthopaedic Surgery, University of California Irvine School of Medicine, Irvine, USA

**Keywords:** intramedullary nail, orthopedic intervention, recurrent fracture, plate fixation, humeral shaft fracture, pycnodysostosis

## Abstract

Pycnodysostosis (PYCD) is an autosomal recessive lysosomal storage disorder of the bone which leads to stereotypical abnormalities consisting of, but not limited to, sclerotic and fragile bone, shortened distal phalanges, and obtuse mandibular angle. Current literature describes the otolaryngological manifestations and treatment of this disorder; however, the treatment of orthopedic fractures in PYCD patients is seldom described and remains a controversial topic. We aim to systematically review the current evidence regarding the optimal treatment of PYCD patients with fractures.

We performed a literature search using PubMed, MEDLINE, Web of Science, and Google Scholar databases. Elig­ibility criteria consisted of English-language literature of PYCD patients undergoing treatment for orthopedic surgery fractures. Non-English papers or literature focused on maxillofacial manifestations/treatment were excluded.

The database search resulted in the identification of 500 articles. After removing duplicates and enforcing our inclusion criteria, 29 case reports/series (40 patients) were included. The average age was 31.25 (­±18.2) years, with 57.5% of patients being female. Overall, 62.5% of patients had consanguineous parents. Additionally, 86.2% reported a history of previous fractures while 47.5% reported a spontaneous or minor trauma fracture, with most fractures occurring in the femur (60.0%) and tibia (40.0%). Radiographic features consisted of densification in the femur 45.0% (18/40), tibia 37.5% (15/40), and spine 25.0% (10/40). Overall, 84.2% of patients were treated with surgical management consisting of internal plate fixation (IPF) (48.3%), intramedullary fixation (20.7%), and Ilizarov external fixation (IEF) (13.8%). Overall, the refracture rate was 25.0% and was lowest in intramedullary fixation (0/6), compared to IPF (3/14) and IEF (3/4). Average time until refracture was 40.6 months (3-132 months).

Long-term follow-up is recommended in patients with PYCD due to the propensity for fractures/refractures. While this study provides the groundwork for the treatment of PYCD patients, further research with higher-evidence studies should be conducted to establish the optimal orthopedic treatment of this disorder.

## Introduction and background

Pycnodysostosis (PYCD) is a rare autosomal recessive lysosomal storage disorder of the bone which leads to stereotypical abnormalities consisting of, but not limited to, sclerotic and fragile bone, shortened distal phalanges, and loss of mandibular angle. As discussed in the Online Mendelian Inheritance in Man, the genotypic basis of PYCD stems from a mutation in the enzyme Cathepsin K on chromosome 1q21. Cathepsin K is highly expressed in osteoclasts, where it is essential for the degradation of the protein components of the bone matrix. Due to this mutation, the lack of functioning Cathepsin K protein results in normally functioning osteoclasts that demineralize bone but are unable to adequately degrade the organic matrix leading to the accumulation of toxic metabolites [1]. The incidence of PYCD is one in one million and, owing to its autosomal recessive nature, is the highest in consanguineous populations with equal distribution among males and females. A significant manifestation of PYCD is the high propensity for spontaneous or low-trauma fractures and the significant delay in healing which poses a considerable challenge for orthopedic treatment [1,2]. At the time of this systematic review, the published literature describes the otolaryngological manifestations and treatment of this disorder; however, the treatment of orthopedic fractures in PYCD patients is far less commonly described and remains a controversial topic. Our aim is to systematically review the current literature and evidence regarding the optimal treatment of pycnodysostosis patients with fractures.

## Review

Methodology

**Figure 1:**
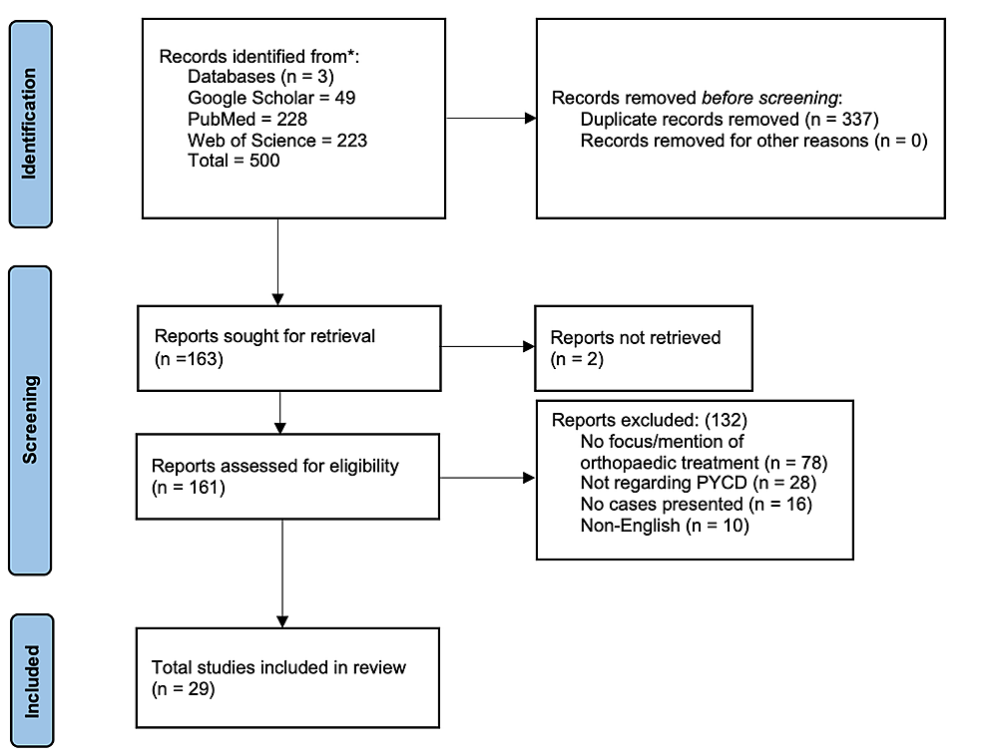
PRISMA diagram detailing the study design and selection process PYCD: pycnodysostosis; PRISMA: Preferred Reporting Items for Systematic Reviews and Meta-Analyses

In December 2021, a literature search to identify all English-language studies focusing on the orthopedic treatment of fractures in PYCD patients was performed on PubMed, MEDLINE, Web of Science, and Google Scholar databases. The keywords searched included the following MeSH terms: “pycnodysost­osis,” “fracture,” and “bone” combined with the Boolean operator “AND” and all synonyms combined with the Boolean operator “OR.” Elig­ibility criteria for this review consisted of English-language literature of pycnodysostosis patients undergoing treatment for orthopedic surgery fractures. Relevant articles were identified, and the results of the search were screened through the title, abstract, and/or full-text article. Non-English papers, literature primarily focusing on the maxillofacial manifestations and treatment, genetic studies, cadaveric studies, and radiological studies were excluded. As shown in Figure 1, The initial research resulted in the identification of 500 articles. In total, 327 of these articles were determined to be duplicates. Of the remaining 173 articles, 29 fit the inclusion criteria of our systematic review.

All the manuscripts were reviewed, and the data were extracted and inputted into an Excel worksheet. The extracted data included author, year, journal, patient age, gender, presenting symptoms and clinical features, fracture location, treatment, follow-up, and clinical outcomes. The data were reviewed by two authors (TT and BL) who agreed on the extracted data. The evidence level of the papers included was discussed and categorized into groups 1-4 based on the method by Wright et al. [3].

Results

Description of the Studies

After applying the inclusion and exclusion criteria, 29 studies consisting of 40 cases were included in the final analysis [2,4-31]. None of the 29 studies were considered level 1 evidence by the criteria described by Wright et al. [3]. All studies, which were case reports and case series, included within this analysis presented level 4 evidence.

Patient Characteristics

Of the 40 patients included in our study, 55.8% of patients were female while 44.2% were male (Table 1). The average age of PYCD patients in our study was 30.37 ­± 17.84 years. Owing to the autosomal recessive nature of the disease, 64.7% of cases with a reported family history had consanguineous parents (11/17). Only 21.9% of patients reported no previous history of fractures while 44.2% (19/40) reported a spontaneous fracture or a fracture caused by minor trauma. It is important to note, that due to the inconsistency in reporting the cause of fracture, the true ratio of fractures caused by minor trauma or spontaneously may differ from our reported results.

**Table 1:** Compiled observations of pycnodysostosis patients with orthopedic fractures found in the literature. *Rate may vary due to inconsistency in reporting the cause of fracture in various case reports.

Characteristics of cases included	Value
Female/Male (%)	57.5/42.5
Average ± standard deviation (years)	31.25 ± 18.2
History of consanguineous parents (%)	62.5
History of previous fractures (%)	86.2
Fracture cause	
Minor trauma/Spontaneous fracture (%)	47.5*

Radiographical Features

Of the patients included in the study, 16.3% (7/40) reported open cranial sutures compared to 4.7% (2/40) reporting closed cranial sutures. Additionally, densification was a common feature reported in the femur (44.2%, 19/40), tibia (34.9%, 15/40), and spine (23.3%, 10/40). Anterior or posterior notching of the vertebra was also reported in 11.6% or five of the 40 cases. Lastly, acro-osteolysis of distal phalanges was a common feature reported in 41.9% (18/40) of the cases (Table 2).

**Table 2:** Rate of presentation of orthopedic radiographic features in cases within the literature.

Radiographical features	Presentation rate
Cranial sutures	
Open	17.5
Closed	5.0
Tibia densification	37.5
Femur densification	45.0
Spine densification	25.0
Spine anterior/posterior notching	12.5
Acro-osteolysis of distal phalanges of the hand	37.5

**Figure 2:**
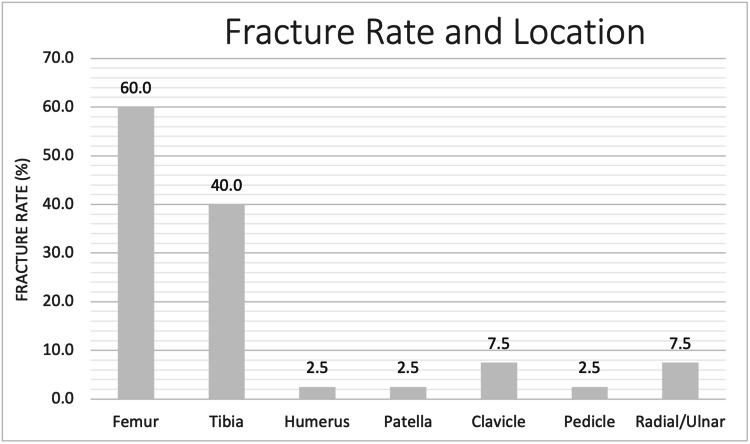
Fracture rate and location of PYCD patients in the included orthopedic-focused PYCD cases in the literature. PYCD: pycnodysostosis

Figure 2 presents the rate and location of fractures in presenting cases in the literature. Of the studies included in our review, femur and tibia were the most common fracture sites with a fracture rate of 55.8% and 37.2%, respectively.

Treatment Approaches

Of the treated PYCD patients, 82.9% were treated with surgical management while 17.1% were treated conservatively (Table 3). Of the 29 patients who reported treatment approaches, the majority 48.3% (14/29) were treated with internal plate fixation while 20.7% (6/29) and 13.8% (4/29) were treated with intramedullary fixation and Ilizarov external fixation (IEF), respectively. The remaining 17.2% (5/29) of patients were treated with other surgical methods such as arthrodesis and K-wire fixation. Of the remaining 11 cases, the studies did not offer greater detail into the type of treatment used, whether surgical or conservative.

**Table 3:** Surgical management and outcomes of pycnodysostosis cases in the literature.

Treatment approach	Value	
Surgical (%)	84.2	
Conservative (%)	18.4	
Rate of specific surgical treatment approach and refracture rate associated	Surgical treatment (%)	Refracture (%)
Internal plate fixation (%)	48.3	21.4
Intramedullary fixation (%)	20.7	0
Ilizarov external fixation (%)	13.8	75.0
Other (e.g., arthrodesis, K-wire fixation) (%)	17.2	-
Overall rate of refracture (%)	25.0	
Average time until refracture with range (months)	40.6 (3–132)	
Median follow-up time with range (months)	24 (2–324)	

The overall refracture rate was 25% (8/32). Refracture rate was the lowest in intramedullary fixation which resulted in zero of the six cases of refracture, while internal plate fixation and IEF resulted in three of 12 cases and three of four cases of refracture, respectively. Average time until refracture was 40.6 months but varied widely with a range of 3-132 months.

Discussion

The heterogeneity of the studies included within this systematic review along with a lack of high-level evidence leads to difficulty in statistically analyzing the data regarding the optimal orthopedic treatment of PYCD. For this reason, a meta-analysis was not performed. 

Despite this constraint, this review accomplished several aims. This systematic review was able to identify the orthopedic presentation of PYCD patients and the typical presenting features aside from the previously well-described stereotypical facies. In the 29 studies with a total of 40 cases, the authors reported high rates of densification in the femur (45.0%), tibia (37.5%), and spine (25.0%) which led to the high fracture risk among PYCD patients. In fact, 88.1% of the included cases within this review endorsed a history of previous fractures, and 44.2% of presenting fractures were reported as spontaneous or caused by minor trauma. An additional feature that was commonly described was acro-osteolysis of distal phalanges which presented in 41.9% of included cases in this review. While these figures can help illuminate the presentation rate of radiographic features of PYCD, the true presentation rate may differ from the rate reported in this study due to the variability in detailed reporting across the cases reports/series in the current literature.

In addition to the orthopedic presentation, it is important to note the facial features consistent with PYCD which can aid in the clinical diagnosis. PYCD consistently presents with specific facial features consistent with a large head due to a prominent bulging of the occiput, micrognathia with an obtuse mandibular angle, exophthalmos, and a narrow palate, along with various other facial features described in previous otolaryngological case reports [31-33].

This systematic review also highlights the current surgical treatment and effectiveness of the orthopedic fractures seen in this patient population. Table 3 presents the rate of surgical treatment approach found within the current literature. Internal fixation with plates and screws was the most common treatment method (35%) and was associated with positive results in several published case reports [2,13,17,24,27,28,34]. Nonetheless, there are intraoperative challenges with increased difficulty in drilling the thick cortex of the bone due to its sclerotic nature, as discussed by multiple authors, including Matar et al. and Yuasa et al. [13,23]. This intraoperative challenge was addressed by Matar et al. by using one drill bit per hole and utilizing constant irrigation with cold saline to decrease the effects of the heat generated [13].

The second most common treatment method for PYCD was intramedullary nail insertion (15%) [7,14,19]. This method presented similar difficulties to the plate fixation as the sclerotic bone hindered the ability to enter the canal [7]. Of note, some studies considered intramedullary nail fixation but, due to the excessive sclerosis of the fracture line, opted for the use of Ilizarov external fixation instead [14]. In a similar case, Marti and Font experienced excessive sclerosis that made it difficult to perform intramedullary nailing, leading to treatment with ankle-joint transfixation which ultimately resulted in nonunion of the tibia [17]. Ultimately, intramedullary nailing and plate fixation both provide sufficient stabilization and alignment of the fracture leading to a lower likelihood of refracture and malunion in the postoperative course.

The third most common treatment method cited in the included studies was IEF (10%). Overall, IEF was most commonly used in cases where attempts at intramedullary nailing were unsuccessful. While a small sample size of four patients exists in the literature for this treatment method, a large proportion (3/4) experienced refractures. This is likely due to the lack of permanent fixation in IEF, as opposed to intramedullary or plate fixation, which jeopardizes bone integrity after removal of IEF leading to an increased likelihood of refractures, as seen in Table 3.

An overall important postoperative complication to note is the likelihood of refracture in these patients after treatment. Due to the brittle nature of the bones in PYCD patients, 25% of treated fractures present with refracture at an average of 40.6 months (3-132 months) after treatment. This highlights the importance of long-term follow-up in this population due to the high likelihood of refracture along with the already high propensity for experiencing other fractures. Additionally, malunion and persistent lucency are other important postoperative outcomes to consider and assess when following up with PYCD patients. Postoperative infection is another risk that was reported in one case by Nakase et al. at the two-week postoperative follow-up and was treated with debridement and irrigation without intramedullary nail removal. While consistent data regarding the risk of postoperative infection rate in PYCD patients are not available, the authors predict an increased risk of infection due to the delay in fracture healing and the propensity for refractures.

Limitations

This systematic review has several limitations regarding the studies included in the review. The conclusions deduced within the study are limited by the retrospective design, highly variable level of detail, and low level of evidence of the included studies. Additionally, the follow-up time reported across the studies included within this review is highly variable and may have led to unreported refractures in PYCD patients leading to a higher true refracture rate than reported within this review. Despite these limitations, our study presents a unique comprehensive review of the current orthopedic treatment of fractures in PYCD patients and lays the groundwork for further research and understanding of this patient population and their optimal surgical treatment.

## Conclusions

Ultimately, surgical management of fractures in PYCD should prioritize proper physiological alignment while providing permanent long-term support to decrease the risk of refractures and malunions. Treatments that prioritize long-term fixation, such as intramedullary nailing or internal plate fixation, offer continuous support for the delayed bone healing in PYCD. Furthermore, secondary to the slow bone healing, long-term follow-up is crucial to monitor treatment success or if alternative treatment is necessary. Despite the data presented within this systematic review, the level of evidence in the included studies is low. Additionally, studies within this review contain high variability in detail of treatment, clinical features, and treatment approach which may alter the true results of the review. Therefore, further studies should be performed with high-quality comparative studies to establish the optimal treatment.
